# Traducción y adaptación transcultural al contexto español del *Person-Centred Practice Inventory-Care* para pacientes

**DOI:** 10.23938/ASSN.1099

**Published:** 2024-12-21

**Authors:** Mónica Vázquez-Calatayud, Ana Choperena, Begoña Errasti-Ibarrondo, Marta Lizarbe-Chocarro, Yvonne Gavela-Ramos, Virginia La Rosa-Salas, Brendan McCormack, María José Galán-Espinilla, Ana Carvajal-Valcárcel

**Affiliations:** 1 Clínica Universidad de Navarra Área de Desarrollo Profesional e Investigación en Enfermería Pamplona España; 2 Universidad de Navarra Facultad de Enfermería Grupo de investigación ICCP-UNAV-Innovación para un cuidado Centrado en la Persona Pamplona España; 3 Instituto de Investigación Sanitaria de Navarra (IdiSNA) Pamplona España; 4 Servicio Navarro de Salud-Osasunbidea Hospital Universitario de Navarra Pamplona España; 5 Universidad de Navarra Facultad de Filosofía y Letras Instituto de Lengua y Cultura Españolas (ILCE) Pamplona España; 6 University of Sydney Faculty of Medicine and Health Susan Wakil School of Nursing and Midwifery Camperdown Australia; 7 Servicio Navarro de Salud-Osasunbidea Gerencia de Atencion Primaria Centro de Salud Ultzama Navarra España

**Keywords:** Adaptación transcultural, Práctica centrada en la persona, Cuidado centrado en la persona, Paciente, Encuestas y cuestionarios, Person-Centered Practice, Person-Centered Care, Cross-cultural adaptation, Patients, Surveys and questionnaires

## Abstract

**Fundamento:**

El cuidado centrado en la persona (CCP) influye positivamente en la persona, mejorando sus habilidades de autocuidado, autonomía, bienestar, calidad de vida y satisfacción con su experiencia de cuidado. El *Person-Centred Practice Inventory-Care* (PCPI-C) es un cuestionario que sirve para evaluar la percepción que los usuarios del sistema sanitario tienen sobre el CCP recibido. El objetivo de este estudio es obtener la primera versión del PCPI-C traducida y adaptada a nuestro contexto español.

**Método:**

La traducción y adaptación transcultural del cuestionario PCPI-C siguió la guía *Translation and Cultural Adaptation of Patient-Reported Outcomes Measures-Principles of Good Practice,* que incluye, además, una sesión cognitiva con un panel de expertos y con un grupo de usuarios de servicios de salud. También se evaluó la claridad y relevancia por ítem mediante el índice de validez de contenido por ítem y para el total del instrumento.

**Resultados:**

No se encontraron dificultades para llegar a un consenso en la traducción de los términos. Por unanimidad, se decidió incluir en el cuestionario la definición del término *compasión* debido a su complejidad y confusión con otras palabras. El índice de validez de contenido por ítem fue excelente en relevancia en los 18 ítems y en claridad en 16. El índice de validez de contenido del instrumento mostró resultados excelentes para claridad y relevancia (≥0,95).

**Conclusiones:**

Se ha obtenido la primera versión del PCPI-C en español adaptada conceptual y semánticamente, y equivalente al cuestionario original, con puntuaciones excelentes en claridad y relevancia.

## Abstract (en)

**Background:**

Person-centered care (PCC) positively impacts individuals by enhancing self-care, autonomy, well-being, skills, quality of life, and satisfaction with their care experience. The Person-Centered Practice Inventory-Care (PCPI-C), grounded in the Person-Centered Practice theoretical framework, is designed to assess healthcare users’ perceptions of the PCC they receive. This study aims to develop the first Spanish version of the PCPI-C, translated and adapted to the Spanish context.

**Methods:**

The translation and cultural adaptation process followed the “Translation and Cultural Adaptation of Patient-Reported Outcomes Measures: Principles of Good Practice” guideline, which included a session with a group of experts and healthcare users. The clarity and relevance of each item, as well as the whole instrument, were assessed using the content validity index.

**Results:**

No difficulties were found during the process. The committee unanimously agreed to include the definition of compassion in the instrument due to its complexity and potential confusion with other terms. Regarding clarity and relevance, the content validity index revealed excellent results for all 18 items in terms of relevance and for 16 items in terms of clarity. The overall validity index for the instrument showed excellent result for both clarity and relevance (≥0.95).

**Conclusions:**

The first Spanish-adapted version of the PCPI-C, conceptually and semantically aligned with the original, is now available and demonstrates excellent scores for both clarity and relevance.

## INTRODUCCIÓN

El cuidado centrado en la persona (CCP) es un modo de ver a la persona de manera individual y trascendente, que vive en relación con otros y pertenece a un contexto familiar y social con unas creencias y valores dentro de una cultura^1^^,^^2^. Esto ayuda a ver a la persona con unas características personales y únicas que la diferencian de las demás, con una historia pasada que continúa en el presente y se proyecta hacia el futuro^1^^,^^2^. Es un enfoque de cuidado holístico de gran relevancia para los sistemas sanitarios, alineado con las prioridades de las políticas sanitarias a nivel mundial^3^^-^^5^. Además, promueve una relación cercana entre el profesional sanitario y el usuario dentro de un entorno propicio para dicha atención^3^^,^^6^. La Organización Mundial de la Salud (OMS) señala que los sistemas de salud centrados en las personas tienen el potencial de generar importantes beneficios, como el aumento de autonomía, el bienestar, la calidad de vida y la satisfacción con el cuidado recibido^5^^,^^7^^-^^11^. Además, se ha demostrado que el CCP puede reducir el número de hospitalizaciones^12^, con la consiguiente reducción de los costes sanitarios asociados^5^^,^^12^.

Debido a que el CCP es esencial para la asistencia sanitaria, a menudo se ha focalizado en diferentes marcos y modelos que incluyen conceptos singulares del CCP^13^^-^^15^, como la atención centrada en la relación^13^ o la atención compasiva^14^. Sin embargo, ninguno de ellos integra los principales atributos que ayudan a comprender a la persona como un todo. El marco de práctica centrada en la persona (PCPF) desarrollado por McCormack y McCance^16^, es un marco teórico reconocido internacionalmente que ayuda a los profesionales sanitarios a comprender todas las dimensiones que integran el enfoque centrado en la persona y cómo estas dimensiones pueden operacionalizarse en la práctica^17^. Este marco se aplica desde hace más de una década en el ámbito sanitario, y aunque originariamente se diseñó para la práctica de la enfermería^18^, actualmente se aplica en los sistemas sanitarios de forma más amplia^1^^,^^16^^,^^17^. El marco PCPF, aplicable en diversos contextos, ha sido recientemente traducido y adaptado al contexto español^19^. Se estructura en cinco dominios (Anexo I): prerrequisitos (atributos requeridos de los profesionales que proporcionan el cuidado), entorno de la práctica (contexto en el que se experimenta el cuidado), procesos centrados en la persona (vías necesarias para interactuar con la persona), y cultura saludable (para desarrollar un cuidado centrado en la persona como resultado); el marco se encuentra dentro del quinto dominio, *macro contexto* (factores de naturaleza estratégica y política que influyen en el desarrollo de culturas centradas en la persona). Por lo tanto, para llegar al centro del marco (cultura saludable) hay que atender a los marcos de referencia estratégicos y políticos, tener en cuenta los atributos del personal como requisito previo para gestionar el entorno de la práctica y para comprometerse eficazmente en los procesos centrados en la persona.

Existen herramientas basadas en marcos teóricos de CCP que evalúan la percepción de los usuarios sobre el proceso asistencial (*Patient-Reported Experience Measures*, PREMs) y sobre los resultados de salud (*Patient-Reported Outcome Measures*, PROMs)^20^^-^^22^. A pesar de que es relevante que existan herramientas que evalúen también la perspectiva de los profesionales de la salud para promover un entorno laboral saludable, son escasos los instrumentos que incluyen ambas perspectivas^22^^-^^24^. Por ejemplo, el *Person-centred Coordinated Care Experience Questionnaire* (P3CEQ), diseñado en el Reino Unido para evaluar las experiencias de atención de personas con enfermedades crónicas, cuenta con un enfoque prioritario en la coordinación asistencial y en la atención centrada en la persona^22^. La escala *Individualized Care Scale (ICS)*, de origen finlandés^23^^,^^25^, carece de dimensiones que consideren la relación interprofesional y el entorno en el que se brinda el cuidado^26^. El *Generic Person-centred Care Questionnaire* (GPCCQ), desarrollado en la Universidad de Gotemburgo^24^, evalúa la relación emocional entre el usuario y el personal de salud, centrándose en aspectos como la participación del usuario en su cuidado y la toma de decisiones compartida. Además, el *Person-Centred Practice Inventory-Care* (PCPI-C) incluye las circunstancias personales del usuario y su entorno familiar^27^; está basado en un marco teórico (PCPF) y evalúa cómo los usuarios perciben el CCP y está diseñado para poder compararse con las percepciones de los profesionales de salud^17^, e incluso con las de los estudiantes^6^.

Por tanto, tener disponible la traducción, adaptación cultural y la validación del PCPI-C en español será un avance crucial para evaluar el CCP en nuestro contexto, dado que permitirá obtener una visión holística del cuidado desde la perspectiva tanto de los usuarios, como de los profesionales y los estudiantes, usando un mismo marco teórico. De este modo, contribuirá a mejorar la calidad del cuidado y promover una cultura centrada en la persona.

El objetivo de este estudio es obtener la primera versión en español del instrumento PCPI-C, traducida y adaptada a nuestro contexto cultural.

## MÉTODO

### Diseño

Se realizó un estudio de adaptación transcultural para obtener la equivalencia conceptual, semántica y de contenido del instrumento *Person-Centred Practice Inventory-Care* (PCPI-C). Para ello, se siguieron los pasos del marco *Translation and Cultural Adaptation of PREMs - Principles of Good Practice (PGP)*, desarrollado por el grupo internacional *Translation and Cultural Adaptation group of International Society for Pharmacoeconomics and Outcome Research´s Quality of Life Special interest Group*^28^.

### Participantes

Las características y el papel desempeñado por las personas involucradas en las distintas fases del proceso de adaptación transcultural se muestran en la Tabla 1.

**Tabla 1:** Participantes y sus roles en la adaptación transcultural del cuestionario *Person-Centred Practice Inventory Care (PCPI-C)*

Persona responsable	Fases	Formación y experiencia
Autor del cuestionario		
BMcC	1,5,6	Experto en el CCP.
Coordinadora del proyecto y Persona de contacto en nuestro contexto español		
ACV	1,3,5,6, 8,10	Nativa española.
		Nivel fluido de inglés.
		Residente en España.
		Ámbito de la salud.
		Familiarizada con el CCP.
		Experta en validaciones psicométricas.
		Experiencia en traducción de cuestionarios.
Personas nativas españolas que actuaron como traductoras		
MVC	2,3,5,8	Nivel fluido de inglés.
		Familiarizada con el CCP.
YGR	Nivel fluido de inglés.	
	Traductora profesional.	
	No familiarizada con el ámbito sanitario ni con el CCP.	
Investigadora nativa española del comité de conciliación		
ACV	3,5,8	Nivel fluido de inglés.
Traductores profesionales nativos ingleses		
CO	4	Nivel fluido de castellano.
AH		No familiarizado con el ámbito sanitario ni con el CCP.
Panel de expertos		
BEI	7	Enfermera con grado de doctor.
		Ámbito de la gestión sanitaria.
		Familiarizada con el CCP.
MJGE	Enfermera con grado de máster.	
	Ámbito de la gestión sanitaria.	
	Familiarizada con el CCP.	
MLCh	Enfermera con grado de doctor.	
	Ámbito clínico-académico.	
	Familiarizada con el CCP.	
	Experta en validaciones psicométricas.	
VLS	Enfermera con grado de doctor.	
	Ámbito académico.	
	Familiarizada con el CCP.	
RGG	Enfermera con grado de máster.	
	Ámbito clínico.	
	Familiarizada con el CCP.	
RAN	Enfermera con grado de máster.	
	Ámbito clínico.	
	Familiarizada con el CCP.	
Grupo de usuarios del sistema sanitario (*Cognitive debriefing*)		
5 participantes	7	3 mujeres y 2 hombres.
		Media de edad: 64,8 años (DE=12,7).
		Educación secundaria (n=2) y terciaria (n=3).
		Ámbito ambulatorio.
Experta en humanidades		
AChA	9	Familiarizada con el CCP.
Prueba piloto		Experiencia en correcciones gramaticales y lingüísticas.
7 participantes	7	4 mujeres y 3 hombres.
		Media de edad: 68,2 años (DE= 13,2).
		Educación secundaria (n=4) y terciaria (n=3).
		Ámbito ambulatorio.

DE: desviación estándar.

### Instrumento de evaluación

El PCPI-C evalúa la percepción que tienen los usuarios sobre el CCP que han recibido durante su contacto con el sistema sanitario^27^. Consta de 18 ítems con respuesta tipo Likert sobre el nivel de acuerdo de la persona con los cinco constructos del dominio *Procesos centrados en la persona* del PCPF. (1=totalmente en desacuerdo, 2=desacuerdo, 3=neutral, 4=de acuerdo y 5=totalmente de acuerdo). Estos constructos son: 1) Trabajar con las creencias y valores de la persona (ítems números 1, 6, 7 y 12); 2) Toma de decisiones compartida (ítems 3, 10, 15 y 18); 3) Implicación auténtica (ítems 9, 11, 16); 4) Estar presente con pleno reconocimiento del otro (ítems 2, 5 y 14) y 5) Trabajar de manera holística (ítems 4, 8, 13 y 17) (Anexo I). El instrumento original, creado en idioma inglés, cuenta con adecuados resultados psicométricos de fiabilidad y validez: consistencia interna de la escala α=0,74 y 0,66-0,79 por constructos^27^. La validez de constructo mostró una estructura de cinco factores, confirmados posteriormente con un adecuado ajuste del modelo, mediante análisis confirmatorio.

### Procedimiento

Se siguieron los diez pasos del marco *Translation and Cultural Adaptation of Patient Reported Outcomes Measures - Principles of Good Practice* (PGP), a excepción de la fase 6 (*Armonización*), puesto que no existían otras versiones del PCPI-C en otros idiomas, solo la versión original inglesa.

1. *Preparación*: la coordinadora del proyecto contactó con uno de los autores del instrumento para informarle del proyecto y solicitar su permiso para realizar la traducción y adaptación transcultural.
2. *Traducción*: dos personas nativas españolas con nivel fluido en inglés, una de ellas traductora profesional, tradujeron de manera independiente el cuestionario del inglés al español, obteniéndose dos versiones españolas (VE_1_ y VE_2_).
3. *Conciliación*: un comité de investigadores (las dos traductoras de la fase anterior y la coordinadora del proyecto) analizó y discutió las ambigüedades y diferencias de cada ítem, obteniendo por consenso la nueva versión española del instrumento (VE_3_).
4. *Retro traducción*: los dos traductores profesionales ingleses tradujeron la nueva versión española consensuada del instrumento (VE_3_) al inglés de manera independiente, obteniendo las versiones VI_1_ y VI_2_.
5. *Revisión de la retro traducción*: el comité de la fase 3 comparó las versiones VI_1_ y VI_2_ y consensuó la nueva versión inglesa del cuestionario (VI_3_), que fue revisada y comparada con la versión original por el autor del cuestionario. Tras finalizar esta fase se preguntó al comité por su percepción durante el proceso de traducción.
6. *Sesión cognitiva* (c*ognitive debriefing)*: en ella participaron un panel de seis expertos y un grupo de cinco usuarios del sistema sanitario. Se les presentó la VE_3_ para que la cumplimentaran. También se solicitó que evaluaran cada ítem según su claridad y relevancia mediante una escala Likert de cuatro puntos (1=no relevante/no claro, 2= algo, 3= bastante; 4= muy relevante/muy claro). Además, para los ítems con puntuaciones 1 o 2 en relevancia o claridad, se solicitó que explicaran y detallaran el motivo, y propusieran sugerencias para su mejora o alternativas en la redacción. Posteriormente, se realizó una puesta en común para especificar los motivos de esas puntuaciones y justificar las discrepancias encontradas.
7. *Revisión de los resultados de la fase 7*: la claridad y relevancia se analizó cuantitativamente mediante el índice de validez de contenido por ítem (I-CVI) y el índice de validez de contenido de todo el cuestionario (S-CVI/Ave). Posteriormente, se analizaron cualitativamente los comentarios y aportaciones realizadas por el panel de expertos y el grupo de usuarios. Se consultó al autor del cuestionario sobre aspectos que crearon duda y necesitaron ser clarificados.
8. *Corrección gramatical de la nueva versión*: la investigadora experta en humanidades corrigió los errores léxico-semánticos (palabras), morfosintácticos (concordancia gramatical de género y número, modo y tiempo verbal) y ortográficos de la última versión del cuestionario, que fueron consensuados con la coordinadora del proyecto.
9. *Informe final*: la coordinadora del proyecto escribió el informe final incluyendo los participantes, las metodologías utilizadas y las decisiones tomadas en todo el proceso.

### Análisis de datos

La variable cuantitativa edad se describió con la media y la desviación estándar (DE). El I-CVI se calculó dividiendo el número de observaciones puntuadas con 3 o 4 entre el número total de observaciones; se calculó el índice Kappa modificado para evitar la posibilidad de coincidencia entre expertos debida al azar^29^, cuyos resultados se interpretaron según la clasificación de Cicchetti y Sparrow (0,40-0,59: aceptable; 0,60-0,74: bueno; ≥0,75: excelente)^30^. El S-CVI/Ave se calculó como la media de I-CVI, interpretándose según Waltz (≥0,90: excelente)^31^. Los comentarios sugeridos por los grupos de expertos y usuarios se evaluaron cualitativamente mediante un análisis de contenido^32^.

### Consideraciones éticas

El estudio fue aprobado por el Comité de Ética de Investigación (CEI, número 2021.125) de la Universidad de Navarra. Antes de iniciarlo, se obtuvo el permiso del autor del cuestionario PCPI-C para realizar la traducción y adaptación transcultural. Todos los expertos y usuarios del sistema sanitario que formaron parte de la investigación dieron su consentimiento para participar en el estudio tras explicarles el objetivo y diseño del estudio de forma oral y por escrito. Los datos de participantes y los resultados obtenidos se trataron de manera confidencial.

## RESULTADOS

Se obtuvieron los resultados detallados a continuación tras seguir las diez fases indicadas:

1. Preparación: Se obtuvo el consentimiento del autor del cuestionario para realizar la traducción y adaptación transcultural del PCPI-C, quien participó activamente durante todo el proceso.

2. Traducción: Se obtuvieron las versiones españolas VE_1_ y VE_2_. El tiempo promedio empleado en la traducción fue de media hora. Las traductoras comentaron que no encontraron dificultades a la hora de traducir el cuestionario, destacando la claridad de los ítems y la sencillez del lenguaje empleado.

3. Conciliación: El comité de investigadores revisó detalladamente las dos versiones españolas VE_1_ y VE_2_, sin apreciar divergencias significativas entre ellas. Concluyeron que ambas eran similares, por lo que no hubo dificultades para llegar a un consenso en ninguno de los 18 ítems del cuestionario, consensuando la VE_3_.

4. Retro traducción: Las versiones inglesas VI_1_ y VI_2_ se obtuvieron de manera conceptual (en vez de literal) y de forma independiente a partir de la versión española consensuada (VE_3_). Ninguno de los traductores refirió dificultades en el proceso. El tiempo promedio requerido fue de unos veinte minutos.

5. Revisión de la retro traducción: El comité de la fase 3 comparó las versiones VI_1_ y VI_2_ y obtuvo la VI_3_ sin dificultades. Esta versión fue revisada por el autor del cuestionario que matizó dos aspectos:

- ítem 4: propuso mantener *home environment* en vez de *family environment*, ya que el primer término era más amplio e incluía también el entorno físico.

- ítem 8: propuso revisar la traducción de *staff ask me about* porque el significado era diferente a *health staff show interest,* por lo que se revisó la traducción española VE_3_ y se consensuó traducir el ítem de la siguiente manera *El personal se interesa por mi vida* para ser más precisos en su significado.

7. Sesión cognitiva: Los motivos de las puntuaciones y las principales discrepancias encontradas se discutieron en una puesta en común grupal que tuvo una duración aproximada de dos horas.

8. Revisión de los resultados de la sesión cognitiva: *Claridad*: todos los ítems obtuvieron I-CVI excelentes excepto los ítems 2 y 7, con I-CVI buenos. El I-CVI/Ave fue 0,94. *Relevancia*: todos los ítems obtuvieron I-CVI excelentes, siendo el I-CVI/Ave 0,97 (Tabla 2).

**Tabla 2:** Claridad y relevancia de la versión española del cuestionario *Person-Centred Practice Inventory-Care* (PCPI-C)

Ítem	Dominio PCP-F: El personal me involucra en la toma de decisiones (*tienen en cuenta mi opinión, preferencias, etc*.) acerca de mi cuidado	Claridad			Relevancia		
		I-CVI	k*		I-CVI	k*	
Trabajar con las creencias y los valores de la persona							
1	El personal sanitario se esfuerza por comprender lo que es importante para mí	1	1	E	0,91	0,91	E
6	Me siento capaz de decir al personal sanitario lo que es importante para mí	0,91	0,91	E	1	1	E
7	Me siento capaz de transmitir al personal mi experiencia de cómo he sido cuidado	0,73	0,7	B	1	1	E
12	El personal me cuida teniendo en cuenta el conocimiento que tiene sobre mí como persona	0,91	0,91	E	0,91	0,91	E
Toma de decisiones compartidas							
3	El personal me involucra en la toma de decisiones acerca de mi cuidado	1	1	E	1	1	E
10	El personal comprueba si tengo toda la información que necesito	1	1	E	1	1	E
15	El personal me ayuda a expresar mis preocupaciones sobre el tratamiento y el cuidado que recibo	1	1	E	1	1	E
18	El personal me ayuda a establecer objetivos realistas	1	1	E	1	1	E
Implicación auténtica							
9	El personal conecta conmigo como persona	0,91	0,91	E	1	1	E
11	Cuando no coincidimos en algún aspecto sobre mi cuidado, el personal intenta llegar a un acuerdo conmigo	1	1	E	1	1	E
16	El personal me escucha y tiene en cuenta mi opinión sobre el cuidado que recibo	1	1	E	0,91	0,91	E
Estar presente con pleno reconocimiento del otro							
2	El personal tiene en cuenta mi experiencia personal para establecer una relación conmigo	0,73	0,7	B	1	1	E
5	El personal me dedica toda su atención cuando recibo su cuidado	1	1	E	1	1	E
14	Cuando me siento disgustado o triste el personal sanitario me cuida de forma compasiva^2^	1	1	E	1	1	E
Trabajar de manera holística							
4	El personal tiene en cuenta mi entorno familiar para cubrir mis necesidades de cuidado	1	1	E	1	1	E
8	El personal se interesa por mi vida	0,82	0,81	E	1	1	E
13	Me siento cuidado/a	1	1	E	1	1	E
17	El personal tiene en cuenta mis circunstancias familiares cuando me cuida	1	1	E	0,91	0,91	E
		S-CVI/Ave = 0,95			S-CVI/Ave = 0,97		

I-CVI: *Item-level content validity index*; k*= índice *kappa* modificado ajustado por azar (0,40-0,59: aceptable; 0,60-0,74: bueno; ≥0,75: excelente)^30^; S-CVI/Ave: promedio de índice de validez de contenido para toda la escala (≥0,90: excelente)^31^; E: evaluación excelente; B: evaluación buena.

Durante el análisis de contenido de las propuestas y sugerencias del panel de expertos y usuarios, estas se aceptaron o desestimaron según las indicaciones del autor del cuestionario y del investigador principal (Tabla 3). Un participante (nivel universitario) comentó que el cuestionario estaba claro y era relevante en general, pero que no estaba seguro de que usuarios con diferente nivel cultural fueran capaces de cumplimentarlo. Además, recalcó que es importante tener en cuenta que este instrumento refleja la percepción de cómo ha sido cuidado la última vez (como indican las instrucciones del instrumento) en vez de su percepción a lo largo del tiempo.

**Tabla 3:** Revisión de los resultados de la sesión cognitiva (fase 8)

Ítem. Definición original	Sugerencias aportadas (por quién)	Decisión final
2. El personal tiene en cuenta mi experiencia personal para establecer una relación conmigo	Añadir (1 usuario): El personal tiene en cuenta mi experiencia personal, *deseos, valores, creencias, preferencias, etc*. para establecer una relación conmigo	No se estimó necesario modificarlo, dado que solo una persona sugirió el cambio y ya estaba implícito en la frase.
3. El personal me involucra en la toma de decisiones acerca de mi cuidado	Clarificar (1 usuario): El personal me involucra en la toma de decisiones (*tienen en cuenta mi opinión, preferencias, etc*.) acerca de mi cuidado	No se contempló aclararlo ya que se consideró redundante.
5. El personal me dedica toda su atención cuando recibo su cuidado	Clarificar (1 usuario): El personal me dedica toda su atención (*me mira, está atento, se interesa por cómo estoy*) cuando recibo su cuidado.	No se estimó pertinente porque ya estaba implícito en la frase.
13. Me siento cuidado/a	Añadir (2 expertos): Me siento *bien* cuidado/a	El autor del cuestionario no lo consideró apropiado porque añadir *bien* era un juicio de valor ,y decir *me siento cuidado* daba la opción de sentirse cuidado a diferentes niveles.
14. Cuando me siento disgustado o triste, el personal sanitario me cuida de forma compasiva	Clarificar el concepto *compasión* (todo el panel de expertos)	Debido a sus diferentes connotaciones en español, se decidió por unanimidad incluir su definición.
1, 2, 3, 4, 5, 8, 9, 10, 12, 15, 16, 17 y 18. Todos ellos comienzan con *El personal sanitario*	Se sugirió utilizar *El personal sanitari*o solo en el primer ítem y, empleando la palabra *personal* en el resto para evitar ser reiterativos y que los participantes contestasen de manera mecánica al cuestionario.	

9. Corrección gramatical del cuestionario: La investigadora experta en humanidades no encontró errores en la última versión del instrumento durante la revisión lingüística. Solo sugirió modificar el ítem 13 *Siempre y cuando yo esté de acuerdo se involucra a mi familia en las decisiones sobre mi cuidado* por *Se incluye a mi familia en las decisiones de mi cuidado, solo cuando quiero que sean incluidos*. Sin embargo, no se modificó para intentar mantener la máxima similitud con el instrumento original inglés y porque se consideró que podía cambiar el sentido de la frase.

10. Informe final: La coordinadora del proyecto redactó el informe final sobre el proyecto incluyendo los participantes, la metodología empleada y las decisiones tomadas a lo largo del proceso. Finalmente, se obtuvo la versión definitiva del cuestionario PCPI-C (Tabla 4).

**Tabla 4:** Versión definitiva de la versión española *Person-Centred Practice Inventory-Care* (PCPI-C)

Piense y reflexione sobre el cuidado recibido a lo largo de su experiencia de salud y, por favor, indique el grado de acuerdo o desacuerdo (1*: Totalmente en desacuerdo. 2: En desacuerdo. 3: Neutral. 4: De acuerdo. 5: Totalmente de acuerdo*) con respecto a las siguientes afirmaciones.	
Ítem	Enunciado
1	El personal sanitario se esfuerza por comprender lo que es importante para mí
2	El personal tiene en cuenta mi experiencia personal para establecer una relación conmigo
3	El personal me involucra en la toma de decisiones acerca de mi cuidado
4	El personal tiene en cuenta mi entorno familiar para cubrir mis necesidades de cuidado
5	El personal me dedica toda su atención cuando recibo su cuidado
6	Me siento capaz de decir al personal sanitario lo que es importante para mí
7	Me siento capaz de transmitir al personal mi experiencia de cómo he sido cuidado
8	El personal se interesa por mi vida
9	El personal conecta conmigo como persona
10	El personal comprueba si tengo toda la información que necesito
11	Cuando no coincidimos en algún aspecto sobre mi cuidado, el personal intenta llegar a un acuerdo conmigo
12	El personal me cuida teniendo en cuenta el conocimiento que tiene sobre mí como persona
13	Me siento cuidado/a
14	Cuando me siento disgustado o triste el personal sanitario me cuida de forma compasiva^1^
15	El personal me ayuda a expresar mis preocupaciones sobre el tratamiento y el cuidado que recibo
16	El personal me escucha y tiene en cuenta mi opinión sobre el cuidado que recibo
17	El personal tiene en cuenta mis circunstancias familiares cuando me cuida
18	El personal me ayuda a establecer objetivos realistas

1: Compasión es la sensibilidad que muestra el profesional de la salud para comprender el sufrimiento de otra persona combinada con la voluntad de ayudar y de promover el bienestar con el fin de encontrar una solución a su problema^52^.

### Prueba piloto

Tras finalizar el proceso de traducción y adaptación transcultural, se realizó una prueba piloto con siete participantes para testar el instrumento. Se seleccionaron personas que en los últimos meses hubieran recibido atención médica en el sistema sanitario de Navarra en régimen ambulatorio, por patologías que no denotaban gravedad y de las cuales se habían recuperado. Emplearon 4,3 minutos de media en completarlo y todos comentaron que era fácil de entender y cumplimentar, sin encontrar dificultades de comprensión para valorar cómo habían sido atendidos. La mayoría de participantes (85,7%) destacaron que se trataba de un instrumento claro y conciso, y todos (100%) indicaron que era un cuestionario corto, sencillo y de fácil cumplimentación. Además, mostraron su acuerdo en que los ítems que componían el cuestionario recogían qué era para ellos el CCP y no sugirieron añadir ningún ítem adicional.

## DISCUSIÓN

Se ha obtenido la primera versión española del instrumento PCPI-C traducido y adaptado transculturalmente al español y equivalente a nivel conceptual, semántico y de contenido a la versión original inglesa. Esta es la primera fase para evaluar las propiedades psicométricas del cuestionario y obtener una versión española válida, fiable y adaptada culturalmente a nuestro contexto^33^.

El PCPI-C^27^ y el PCPI-S^17^, este último traducido y adaptado al contexto español^34^, están basados en el marco teórico PCPF^16^^,^^18^. La disponibilidad de estos dos instrumentos, orientados hacia una práctica centrada en la persona y basados en un marco teórico común, permite su aplicación en diversos contextos sanitarios, tanto para profesionales de la salud como para diferentes grupos de usuarios que han tenido contacto con el sistema sanitario. De este modo, además de obtener la percepción del profesional (PCPI-S), se incorpora la visión de la persona atendida por el profesional (PCPI-C), lo cual es esencial para una evaluación multidimensional e integrada del cuidado centrado en la persona dentro del sistema de salud^27^.

Una reciente revisión sistemática indica que el interés por los estudios comparativos culturales ha crecido de manera exponencial en las últimas décadas^35^. Utilizar un cuestionario en una población diferente a aquella para la que fue originalmente diseñado requiere verificar su comparabilidad transcultural a través de una validación lingüística y, posteriormente, psicométrica^36^. Un instrumento que ha obtenido resultados adecuados en una cultura debe examinarse en profundidad para identificar diferencias y similitudes culturales, lo cual implica un proceso más exhaustivo que una simple traducción. Para alcanzar la equivalencia entre el instrumento original y el traducido, el proceso de adaptación debe seguir un enfoque universalista, que reconoce que los conceptos pueden variar entre culturas, tener significados distintos o incluso no existir^37^. Por tanto, asegurar la equivalencia conceptual por ítem, semántica, operacional, de medida y cultural es esencial para asegurar la validez y fiabilidad del instrumento^38^. Dado que el PCPI-C fue desarrollado para la población y el sistema de salud irlandés, y desde la perspectiva de autores irlandeses, es necesario adaptarlo a la cultura española. Esto implica comparar el instrumento original con el contexto español, traducirlo y adaptarlo a nuestra realidad.

A la hora de adaptar un instrumento que ha sido creado para una cultura concreta a otra diferente, no existe un estándar de calidad universal. Por ello, es necesario considerar cuidadosamente la metodología utilizada para asegurar la adaptación transcultural, garantizando que el instrumento se ajuste lo mejor posible a la población diana^35^. La guía empleada para llevar a cabo todo el proceso de adaptación y validación transcultural del PCPI-C, la *Translation and Cultural Adaptation of Patient-Reported Outcomes Measures-Principles of Good Practice (PGP),* se originó a partir de un consenso de más de doce guías previas de traducción y adaptación cultural^28^. Esta guía es ampliamente utilizada a nivel internacional, lo que asegura la rigurosidad y calidad del proceso. En el proceso de traducción del PCPI-C al español participó un comité de tres personas, lo que según Wild y col^28^ es una fortaleza, ya que se evita mantener el estilo de una sola persona y se mejora la comprensión de la traducción obtenida. Además, la participación del autor del cuestionario en el proceso de traducción y validación transcultural del cuestionario PCPI-C, del PCPI-S^34^ y del marco PCPF^19^ garantizan la equivalencia entre las diferentes versiones y las originales y el rigor en todo el proceso^28^.

Evaluar la validez de su contenido es esencial para asegurar la calidad del instrumento y como paso previo a testar una validez más general e inferencial, como la de criterio o de constructo a través de las puntuaciones obtenidas en la población diana^39^. Esta validez debe representar adecuadamente los dominios y la definición operativa del constructo de interés^40^. En la presente investigación, los resultados del análisis de la validez de contenido del instrumento PCPI-C demuestran que el constructo CCP está adecuadamente reflejado en los dieciocho ítems del cuestionario, ya que ninguno de ellos obtuvo un valor inadecuado tras el ajuste a través del cálculo del índice kappa ponderado que ha permitido eliminar la probabilidad de concordancia debida al azar entre la opinión de los expertos. Ello refuerza la importancia de que el proceso de validación no consista solo en la emisión del juicio personal de expertos, sino que además esté reforzado por un respaldo estadístico que permita incrementar la calidad de la validación y reflejar de manera precisa el constructo CCP^41^. Igualmente, añadir comentarios cualitativos a los índices de contenido cuantitativos (I-CVI) refuerza la coincidencia de ambas perspectivas, enriquece los datos y profundiza en la propia validación.

De hecho, en los últimos años ha quedado patente el interés y la importancia de involucrar más activamente a las personas que han hecho uso de los sistemas de salud en los estudios que evalúan los recursos sanitarios y los resultados en salud, como pone de manifiesto la abundante investigación relacionada con los *Patient-Reported Outcome Measurement* (PROMs) y *Patient-Reported Experience Measures* (PREMs)^20^^-^^22^^,^^45^^,^^46^. Así, en el ámbito clínico, los profesionales de la salud pueden utilizar el PCPI-C para favorecer su relación con la persona cuidada y tener la oportunidad de abordar los aspectos que le son relevantes^47^. Los usuarios subrayan la importancia de que los profesionales compartan tiempo con ellos, les informen de manera adecuada y establezcan una relación significativa^20^^,^^41^, elementos del cuidado que están contemplados y ampliados en la presente herramienta PCPI-C. En futuros estudios, además de utilizarse como instrumento para comparar la percepción de cada individuo sobre el cuidado recibido (PCPI-C) y la perspectiva del profesional al proporcionar el cuidado (PCPI-S), se podría explorar su aplicabilidad como herramienta para mejorar la comunicación con el usuario sanitario y su familia.

Una de las limitaciones de este estudio es que se centra solo en el primer paso del proceso de validación. No obstante, este primer paso es imprescindible y esencial para poder proceder con el segundo paso, la evaluación de las propiedades psicométricas del instrumento, fase en la que está inmerso actualmente el equipo investigador. Cabe destacar que esta fase de adaptación transcultural es indispensable para garantizar la adaptación del PCPI-C a un lenguaje y contexto cultural diferente al original^33^ y, por tanto, condiciona el resto de propiedades psicométricas^48^.

Contar con una herramienta adaptada a nuestro contexto que complemente la visión del CCP desde el punto de vista del usuario sanitario, como agente central de una cultura centrada en la persona, permitirá una cuantificación de su experiencia de cuidado como ejemplo de PREM y ayudará a diseñar e implantar estrategias efectivas para el desarrollo del CCP^49^^,^^50^. De este modo, se dará respuesta a la estrategia prioritaria de humanización del Sistema Nacional de Salud y, más concretamente, del Sistema Sanitario de Navarra, que tiene entre sus objetivos sensibilizar a los profesionales sobre la cultura de la humanización, capacitarlos en la adquisición y promoción de competencias en el CCP, y contribuir al desarrollo profesional incluyendo la percepción de las personas que han utilizado los recursos sanitarios, incorporándolos como agentes activos en el proceso de cuidado^51^.

En conclusión, todos los ítems obtuvieron una puntuación excelente para relevancia y 16 de 18 ítems la obtuvieron para claridad; los índices de validez de contenido del cuestionario fueron excelentes (≥95) para ambos aspectos. No se encontraron dificultades para llegar a un consenso en la traducción de los términos pero por unanimidad se decidió incluir la definición de *compasión* debido a su complejidad. Se ha obtenido una versión adaptada al español clara, relevante y conceptualmente equivalente a la versión original del PCPI-C.

## Figures and Tables

**Figure:**
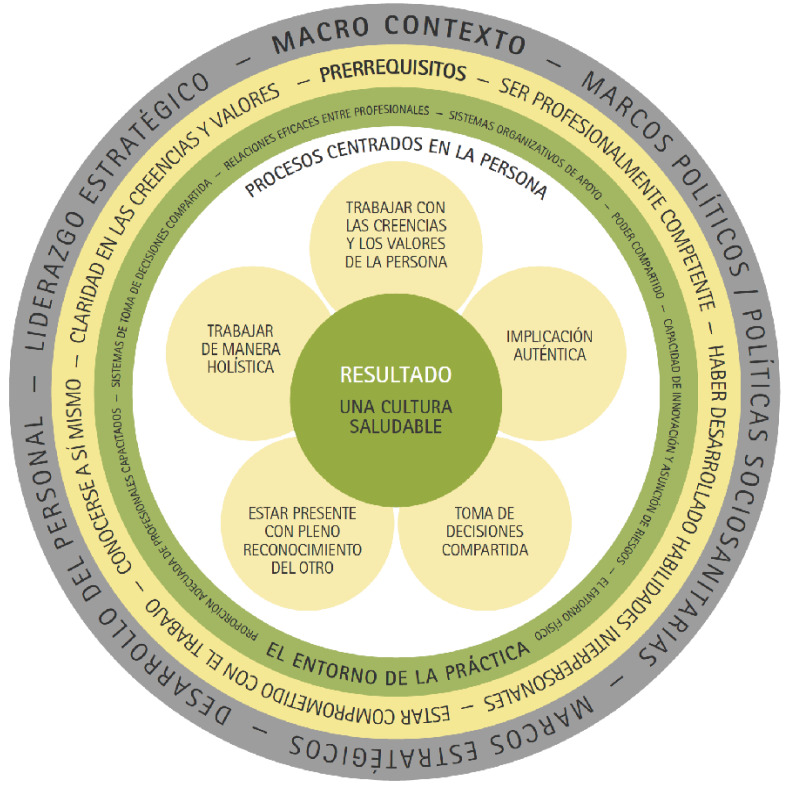
Marco Person Centred Practice Framework (PCPF)*

* Tomado de Choperena et al.^19^. Se dispone de permiso de reproducción.

## Data Availability

Se encuentran disponibles bajo petición al autor de correspondencia.
